# Normal Brain Response to Propofol in Advance of Recovery from Unresponsive Wakefulness Syndrome

**DOI:** 10.3389/fnhum.2016.00248

**Published:** 2016-06-02

**Authors:** Stefanie Blain-Moraes, Rober Boshra, Heung Kan Ma, Richard Mah, Kyle Ruiter, Michael Avidan, John F. Connolly, George A. Mashour

**Affiliations:** ^1^School of Physical and Occupational Therapy, McGill UniversityMontreal, QC, Canada; ^2^Department of Linguistics and Languages, McMaster UniversityHamilton, ON, Canada; ^3^Department of Anesthesia, McMaster UniversityHamilton, ON, Canada; ^4^Department of Anesthesiology, Washington UniversitySt. Louis, MO, USA; ^5^Department of Anesthesiology, University of MichiganAnn Arbor, MI, USA

**Keywords:** consciousness, unresponsive wakefulness syndrome/vegetative state (UWS/VS), propofol, anesthesia, event-related potentials (ERPs), functional connectivity

## Abstract

Up to 40% of individuals with unresponsive wakefulness syndrome (UWS) actually might be conscious. Recent attempts to detect covert consciousness in behaviorally unresponsive patients via neurophysiological patterns are limited by the need to compare data from brain-injured patients to healthy controls. In this report, we pilot an alternative within-subject approach by using propofol to perturb the brain state of a patient diagnosed with UWS. An auditory stimulation series was presented to the patient before, during, and after exposure to propofol while high-density electroencephalograph (EEG) was recorded. Baseline analysis revealed residual markers in the continuous EEG and event-related potentials (ERPs) that have been associated with conscious processing. However, these markers were significantly distorted by the patient’s pathology, challenging the interpretation of their functional significance. Upon exposure to propofol, changes in EEG characteristics were similar to what is seen in healthy individuals and ERPs associated with conscious processing disappeared. At the 1-month follow up, the patient had regained consciousness. We offer three alternative explanations for these results: (1) the patient was covertly consciousness, and was anesthetized by propofol administration; (2) the patient was unconscious, and the observed EEG changes were a propofol-specific phenomenon; and (3) the patient was unconscious, but his brain networks responded normally in a way that heralded the possibility of recovery. These alternatives will be tested in a larger study, and raise the intriguing possibility of using a general anesthetic as a probe of brain states in behaviorally unresponsive patients.

There is increasing evidence that some patients with a diagnosis of unresponsive wakefulness syndrome (UWS; previously referred to as vegetative state) are in a state of “covert consciousness” (Mashour and Avidan, 2013), i.e., conscious but unable to respond to or communicate with others. UWS is defined as a state of complete unawareness of the self and the environment, accompanied by sleep-wake cycles, with either complete or partial preservation of hypothalamic and brain-stem autonomic function (Laureys et al., 2010). In clinical practice, lack of responsiveness is typically attributed to unconsciousness and as a result, up to 40% of patients diagnosed with UWS are actually covertly conscious (Schnakers et al., 2009). Recently, much attention has been given to using neurophysiological data to gain insight into the level of consciousness of behaviorally unresponsive individuals (Bruno et al., 2011). For example, certain event-related potentials (ERPs)—electroencephalographic (EEG) signatures reflecting the brain’s response to stimuli—have been proposed as markers of consciousness (Kotchoubey et al., 2005; Bekinschtein et al., 2009; Harrison and Connolly, 2013). Studies of effective connectivity between brain regions have suggested that impaired feedback/top-down connectivity (e.g., from frontal to temporal cortices) can distinguish presence vs. absence of consciousness (Laureys et al., 1999; Boly et al., 2011; Rosanova et al., 2012). Machine learning techniques that classify neurophysiological patterns in response to motor imagery commands have been proposed as a means of distinguishing patients with covert consciousness from those with UWS (Cruse et al., 2011). However, the ability of these methods to detect covert consciousness has been critiqued on the basis of technical or statistical issues (King et al., 2011; Goldfine et al., 2012; Naccache et al., 2015; Tzovara et al., 2015a,b). Another issue with current approaches is that neural markers must be compared to those of healthy controls using a between-subject study design, and interpretation of the functional significance of their presence or absence can be controversial. General anesthesia offers a potential paradigm for a within-subject design for the detection of covert consciousness. Drugs, that are typically administered to achieve general anesthesia could be administered to behaviorally unresponsive patients, and their neurophysiological data compared before, during and after. Here, we report a pilot of this within-subject approach in a patient with apparent UWS, and examine the effect of the anesthetic drug propofol on neurophysiological patterns.

## Case Report

**Figure 1:**
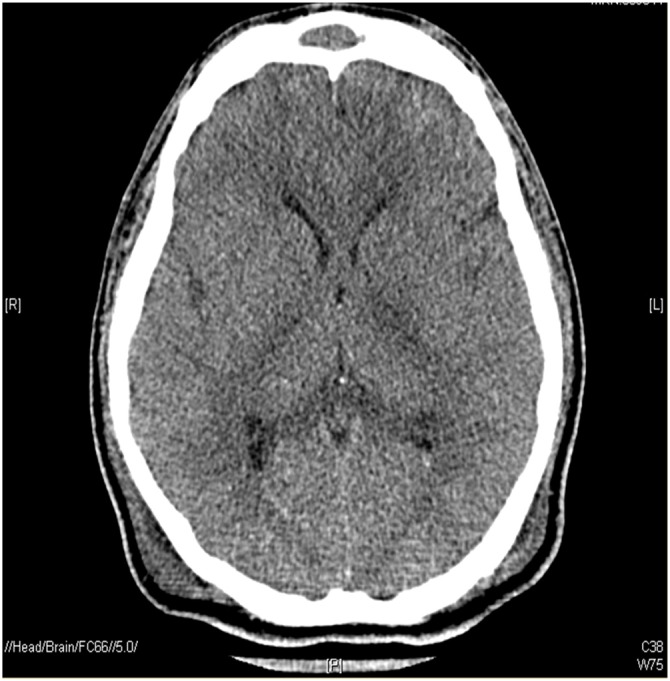
**Patient computed tomography scan recorded 43 days post-trauma**.

A 29-year old male involved in a motor vehicle collision presented at the Emergency Department with multisystem trauma. Computed tomography scans revealed a right parietal subdural hematoma, acute traumatic subarachnoid hemorrhage as well as diffuse axonal injury; signs of increased intracranial pressure, such as sulcal effacement and loss of gray-white differentiation, were also observed (Figure 1).

He was transferred to the intensive care unit, the trachea was intubated and the lungs were mechanically ventilated. The EEG report 21 days post-trauma stated: “This is an abnormal video EEG due to the presence of generalized slowing over the background activity. The above-described frontally dominant alpha-like activity may suggest alpha coma in evolution. Poor prognostic features from the current EEG include the paucity of waveforms as well as the lack of response to multiple afferent stimuli. These findings are in keeping with the clinical diagnosis of diffuse axonal injury.” The patient became involved in this pilot investigation 56 days post-trauma. At that time, his medications did not include any sedatives or barbiturates. Tests of wakefulness and responsiveness were consistent with a diagnosis of UWS (Glasgow Coma Scale = 4; Coma Recovery Scale-Revised = 4). The study was conducted 58 days post-trauma, with the patient demonstrating no significant behavioral changes from day 56. A substitute decision maker provided informed consent for the study, which was approved by the Hamilton Integrated Research Ethics Board.

## Materials and Methods

### Experimental Design

An auditory stimulation series was presented to the patient: (1) at baseline; (2) during steady-state anesthetic infusion; and (3) after drug exposure. The stimulation paradigm was designed to elicit three ERP waveforms (N100, Mismatch Negativity (MMN), P300) and consisted of an auditory oddball series (Morlet and Fischer, 2014) of four sounds: standard tones (80%), deviant tones (14%), participant’s own name (PON; 3%), and unfamiliar novel sounds (NS; e.g., car horn; 3%). Throughout this experiment, an electroencephalogram was acquired from 64 channels using a bandpass of 0.1–100 Hz sampled at 512 Hz, with an active reference.

### Anesthetic Protocol

The anesthetic protocol was developed using a pharmacokinetic simulation program (Tivatrainer), based on the patient’s age, weight, height, sex and American Society of Anesthesiology physical status. The customized pharmacokinetic/pharmacodynamic model was used to determine the bolus doses and infusion rates of propofol through the experiment, and the times required to reach the targeted effect site concentrations. Steady-state anesthetic infusion was targeted at 2 mcg/mL, and the post-exposure period began when concentrations dropped below 0.5 mcg/mL.

### Electroencephalographic Analysis

EEG signals were re-filtered to a 0.5 and 50 Hz bandpass and re-referenced to an average reference. Signals were visually inspected to reject epochs and channels with noise or non-physiological artifacts.

#### Continuous EEG Analysis

Spectrograms, topographic power maps and phase-amplitude coupling were calculated according to the methods detailed in Blain-Moraes et al., 2015. For all combinations of channels, functional connectivity was calculated using phase lag index (PLI; Stam et al., 2007) and directed functional connectivity was calculated using directed PLI (dPLI; Stam and van Straaten, 2012). A representation of the EEG network was constructed by selecting the top 30% of PLI values calculated between all combinations of channels; higher-degree nodes were considered hubs of the network.

#### ERP Analysis

Analysis was conducted on eight artifact-free electrode sites where the ERP components of interest are typically observed (F3, Fz, F4, C3, Cz, C4, P3, P4). For each condition (baseline, propofol-exposure and post-exposure), EEG activity was averaged within each of the four stimulus types (standard tone, deviant tone, PON, NS), using data segmented 100 ms prior to 1000 ms after the onset of the auditory stimulus. The standard tone average was examined for the N100, and waveforms in each condition were compared on a point-by-point basis using serial *t*-scores to establish where they differed in time (Perrin et al., 2006). MMNs were sought in the deviant–standard subtraction wave, and P300s were sought in both PON–standard and NS–standard subtraction waves. Waveforms were compared using the same serial *t*-score method, and differences were significant at *p* < 0.05.

## Results

### Patient Retained Neural Pathways Associated with Unconscious Processing

**Figure 2:**
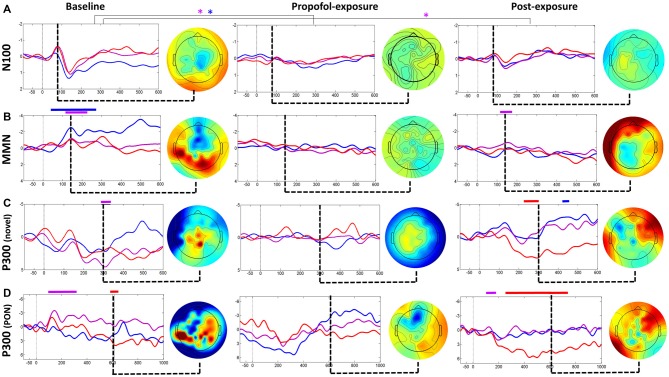
**Event-related potential (ERP) analysis for baseline, propofol exposure and post-exposure periods.** Waveforms **(A)** and subtraction waves **(B–D)** are presented for electrodes Fz (blue), Cz (purple) and C4 (red). Time periods where significant differences occurred are indicated by a color-matched bar above the waveform plot. Blue*, significant differences in Fz waveforms; Purple*, significant differences in Cz waveforms.

ERP analysis at baseline revealed a typical N100-P200 waveform complex (Figure 2A), and a classic frontocentral MMN waveform (Figure 2B). While these ERPs reflect a pre-attentive, non-conscious response that can be frequently detected in individuals with UWS and even coma (Bekinschtein et al., 2009), they demonstrate that this patient retained intact residual higher-order auditory processing. NS generated a frontally-distributed P300 at Cz (i.e., P3a; Figure 2C), which has been associated with unconscious attention (Polich, 2007). Collectively, these ERPs both confirm data quality and demonstrate that the patient retained neural pathways associated with unconscious processing.

### Patient EEG Characteristics are Markedly Different from Healthy Individuals

**Figure 3:**
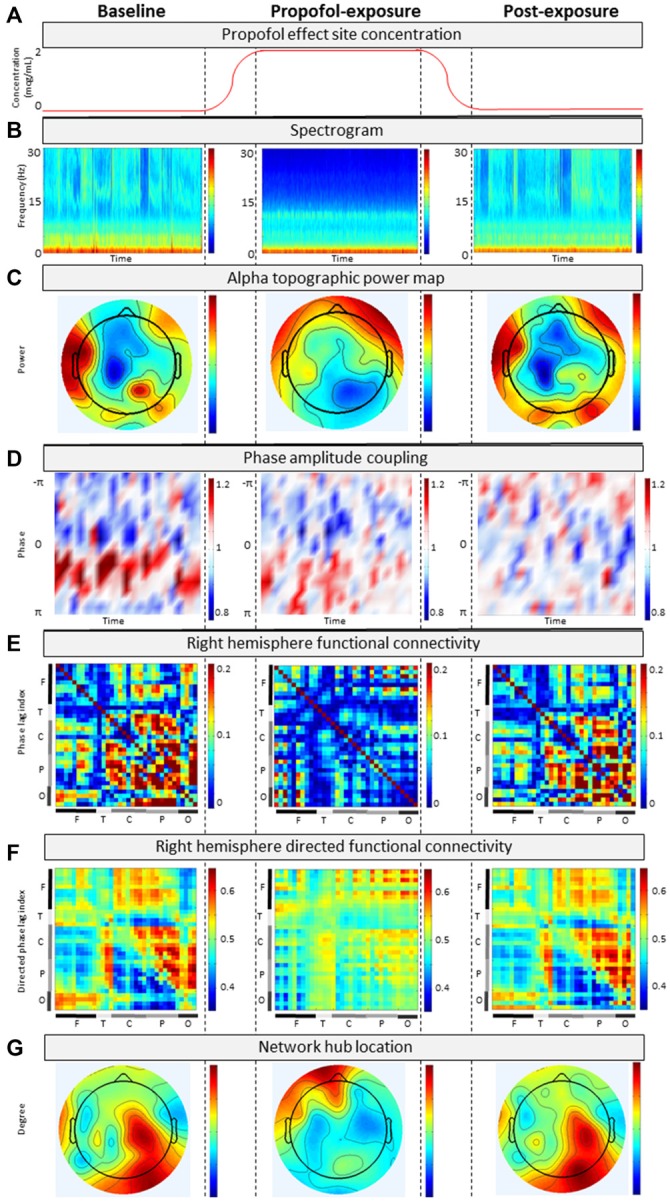
**Analysis of continuous electroencephalograph (EEG) for (A) baseline, propofol-exposure and post-exposure periods using (B) spectrogram; (C) alpha topographic power map; (D) phase-amplitude coupling; (E) phase lag index; (F) directed phase lag index; and (G) network hub location**.

Analysis of the patient’s baseline continuous EEG (Figure 3A) revealed pathological characteristics. Contrary to healthy individuals, the averaged spectrogram over all electrodes showed no peak in alpha power (Figure 3B), and existing alpha power was distributed laterally rather than occipitally (Figure 3C). Phase-amplitude coupling between low-frequency (0.1–1 Hz) and alpha (8–14 Hz) oscillations over parietal channels resembled peak-max patterns, which have been observed in this region in normal unconscious humans (Figure 3D; Mukamel et al., 2014). Functional and directed functional connectivity were atypically asymmetric between left and right hemispheres, with the left hemisphere showing an unusually uniform distribution of PLI and dPLI values between all channels. Anterior-posterior directed connectivity was predominantly observed between centroparietal and centro-occipital regions, as opposed to the frontal-parietal and frontal-occipital connectivity in healthy individuals (Figure 3F). Network hubs were in the parietal area, similar to healthy individuals, but focused over the right hemisphere instead of over the midline (Figure 3G). The ERP response to PON occurred approximately 610 ms post-stimuli with a left parietal and right frontal distribution, as opposed to the typical P300 (i.e., P3b) which occurs approximately 300 ms post-stimulus with a midline-parietal distribution (Figure 2D).

### EEG Changes due to Propofol are Associated with Loss of Consciousness

During propofol exposure (Figure 3A), all EEG characteristics demonstrated significant changes from their baseline patterns. All ERP components disappeared (Figure 2). We observed decreases in high-frequency power (Figure 3B), anteriorization of alpha power (Figure 3C) and loss of phase-amplitude coupling patterns in the parietal channel (Figure 3D). The strong centroparietal and centro-occipital functional and directed functional connectivity observed in the right hemisphere during baseline recording were neutralized by propofol (Figures 3E,F). The direction of information flow became predominantly “feed-forward” (i.e., occipital-to-frontal and parietal-to-frontal) in the anesthetized state. Network hubs shifted from a parietal to a frontal location (Figure 3G). All of these changes have been associated with propofol-induced unconsciousness in healthy volunteers or patients (Lee et al., 2013; Purdon et al., 2013; Blain-Moraes et al., 2015). Most EEG characteristics returned to baseline patterns post-exposure, with the exception of phase-amplitude coupling and the PON-related P300 (Figure 2D).

## Discussion

This report is, to our knowledge, the first to systematically assess neurophysiological signatures with and without general anesthetic administration in a patient diagnosed with UWS. Despite the evidence suggesting a pathological state of unconsciousness and abnormal EEG characteristics associated with the patient’s traumatic injury, propofol caused changes in continuous EEG that have been consistently associated with loss of consciousness in normal brains: anteriorization of alpha rhythms, neutralized functional connectivity between brain regions, suppressed feedback or recurrent processing, and a shift in the primary network hub location from parietal to frontal regions (Lee et al., 2013; Purdon et al., 2013). These changes returned to baseline as the effects of propofol waned. Importantly, upon 1-month follow up after the study, the patient had recovered consciousness clinically (Glasgow Coma Scale = 14; Coma Recovery Scale-Revised = 23), was able to follow commands, and respond verbally in an appropriate manner to conversation.

Recent efforts to assess consciousness using neurophysiological data have been limited by the need to interpret data from unresponsive patients by a comparison to healthy controls (Bekinschtein et al., 2009; Cruse et al., 2011; Goldfine et al., 2012; Naccache et al., 2015; Tzovara et al., 2015a,b). The challenges inherent in interpreting the EEG of unresponsive patients are clearly illustrated in this patient. Pathological patterns in the baseline continuous EEG make it difficult to determine the functional significance of residual neural markers associated with conscious awareness in healthy controls (e.g., anterior-to-posterior directed connectivity, parietal hub locations). Interpretation of baseline ERPs is also complex. The PON-related P300 has a markedly longer latency and altered cortical topography compared to healthy controls, and while it is conceivable this is the result of severe brain damage, we are unable to draw any unequivocal conclusions about this patient’s level of consciousness without additional tools.

This study pilots a novel within-subject paradigm using a general anesthetic to perturb brain networks in a patient diagnosed with UWS. There are three potential explanations for our findings. First, it is possible that this patient was conscious despite the behavioral and EEG signs of unconsciousness. In this framework, the patient’s covert consciousness was suppressed by the administration of propofol. Second, it is possible that the patient was unconscious (i.e., the diagnosis of UWS was correct), but that the previously reported signatures of propofol-induced unconsciousness are in fact a drug-specific phenomenon, and not related to states of unconsciousness, *per se*. Third, it is possible that the patient’s brain state shifted from pathological unconsciousness to another state induced by propofol. This last possibility is interesting in light of the patient’s ultimate recovery, because it suggests that the brain was capable of dynamic reconfiguration after propofol administration despite the apparent pathology associated with the trauma. These opposing explanations will be tested by replicating this case study in a larger cohort of patients with UWS diagnosis (clinical trial NCT02659228). If the techniques proposed in this brief report attenuate residual markers of consciousness in UWS, we would expect approximately 40% of the participants to demonstrate a change from baseline patterns in the anesthetized state (the current rate of misdiagnosis). However, if these are drug-specific rather than state-specific changes, all participants will show similar patterns of attenuation and return to baseline. Finally, if the phenomenon we observed reflected a dynamic shift in brain state from pathological to pharmacological control, this will need to be studied in a larger population with attention to the underlying injury and the outcome. In this case study, the patient recovered, raising the provocative possibility that the normal shifts to neurophysiological signs of propofol-induced unconsciousness revealed a preserved repertoire of brain states that was associated with the ability of this patient to regain consciousness.

## Conclusion

In this report, the neurophysiological signatures of a patient diagnosed with UWS were systematically assessed with and without general anesthetic administration. Propofol induced changes in the patient’s EEG that have been consistently associated with loss of consciousness in normal brains. The results raise the intriguing possibility of using a general anesthetic as a probe of consciousness in behaviorally unresponsive patients.

## Author Contributions

SB-M, MA, JFC and GAM conceived of and designed the study. RB, HKM, KR and RM collected the data. SB-M and RB performed the data analysis. SB-M wrote the first draft of the manuscript, which was edited and approved by all authors.

## Conflict of Interest Statement

The authors declare that the research was conducted in the absence of any commercial or financial relationships that could be construed as a potential conflict of interest.
